# The association between frailty index and abdominal aortic calcification in the middle-aged and older US adults: NHANES 2013–2014

**DOI:** 10.3389/fpubh.2025.1546647

**Published:** 2025-05-07

**Authors:** ZhengJun Zhang, Peng Wu, Shaobin Yang, Baozhen Zhu, Dapeng Chen, Xiaocheng Li, Yarong Wang, Ning Yan

**Affiliations:** ^1^Department of Cardiology, General Hospital of Ningxia Medical University, Yinchuan, China; ^2^The First Clinical College of Ningxia Medical University, Yinchuan, China; ^3^Department of Intervention, Tongxin County People’s Hospital, Wuzhong, China; ^4^Institute of Basic Medical Sciences, Xi’an Medical University, Xi’an, China; ^5^Department of Geriatric, General Hospital of Ningxia Medical University, Yinchuan, China

**Keywords:** subclinical atherosclerosis, vascular calcification, abdominal aortic calcification, frailty index, NHANES

## Abstract

**Background:**

Abdominal aortic calcification (AAC) is one of the earliest observed forms of atherosclerotic calcification and is crucial for early cardiovascular risk prediction. Frailty, a global clinical and public health challenge, is associated with increased risks of mortality, functional decline, and loss of independence. However, the relationship between the Frailty Index (FI) and AAC among middle-aged and older adults has yet to be explored.

**Methods:**

This study analyzed data from 2013 to 2014 National Health and Nutrition Examination Survey (NHANES) cohort, focusing on individuals aged ≥ 40 years. The FI was calculated using a 49-item model to assess frailty status and participants were stratified into three groups: non-frail (FI ≤ 0.15), pre-frail (0.15 < FI ≤ 0.25), and frail (FI > 0.25). AAC was measured by dual-energy X-ray absorptiometry and quantified by Kauppila scores. Severe AAC was defined as an AAC score > 6. The relationship between FI and AAC was investigated using multivariable logistic regression, sensitivity analyses, and smoothing curve fitting. Subgroup analyses and interaction tests were conducted to assess the stability of this association across different populations.

**Results:**

A total of 2,572 participants were enrolled in this study. Following adjustment for potential confounders, FI exhibited a statistically significant positive association with both AAC score (β = 2.64, 95%CI = 1.20–4.08) and Severe AAC (OR = 6.36, 95%CI = 1.48–27.41). Similar trends (*P* for trend < 0.05) were observed when FI was analyzed as a categorical variable. Smooth curve fitting and subgroup analysis were used to investigate the relationship between baseline FI Z-score and AAC score and Severe AAC. Interestingly, we found that the FI Z-score was linearly related to the occurrence of severe AAC, while it was nonlinearly related to the AAC score. The FI-Z score was positively associated with the likelihood of AAC score before the breakpoint (*K* = 0.78), but not significant after the breakpoint. The association between FI-Z score and Severe AAC was stable in the different subgroups (all *P* for interaction > 0.05).

**Conclusion:**

Our study indicated a stable positive correlation between FI and AAC. FI may serve as a biomarker for early subclinical atherosclerosis detection in middle-aged and older US adults.

## 1 Introduction

Cardiovascular disease (CVD) is the leading contributor to mortality and morbidity in both the United States (US) and worldwide, leading to a heavy burden on the medical system (1, 2). The prevention of CVD is a matter of concern within the realms of research and health policy (2). Vascular calcification (VC) refers to a prevalent vascular condition characterized by the anomalous accumulation of minerals within the media or lipoproteins within the intima, is a potential risk factor for the progression and rupture of atherosclerotic plaques and has been highly associated with CVD events (3). It is a common condition in individuals with diabetes and chronic kidney disease (CKD), affecting over 70% of patients (4). Even in the young adults with end-stage renal disease (ESRD) who do not exhibit common cardiovascular risk factors like hypertension or dyslipidemia, this phenomenon still occurs (5). To data, the mechanism of VC is complex and not well-defined, possibly linked to uncontrolled mineralization, homeostasis of calcium and phosphorus levels, imbalance between osteochondrogenic signaling, inflammatory responses, endoplasmic reticulum stress, mitochondrial dysfunction, iron homeostasis, cell apoptosis and degradation of substrates (6). Animal studies have demonstrated the potential efficacy of statins and calcium channel blockers (CCBs) in treating VC, however, their effectiveness in human subjects is still a topic of debate (7, 8). While studies in rat models have demonstrated the efficacy of sodium thiosulfate in treating VC, its clinical application in humans requires verification through more extensive randomized controlled trials to ensure both safety and therapeutic effectiveness (9). Given the association of VC with CVDs and mortality, along with the treatment challenges involved, the prevention and improvement method of VC deserve further exploration.

Abdominal aortic calcification (AAC), a prevalent form of VC, develops earlier than coronary artery calcification (CAC) and has demonstrated the ability to forecast subclinical CVDs and subsequent CVD events, irrespective of traditional risk factors. Data from the Framingham Heart Study demonstrate a sex-specific disparity in AAC prevalence, with males under 45 years exhibiting a 22.4% incidence compared to 16.4% in females (10). In a 13-year prospective cohort study of 2,056 community-dwelling U.S. women, the prevalence of AAC increases with age, from 60% in 65–69 years of age to 96% in 85 years and older (11). A long-term prospective follow-up study indicated that AAC not only serves as a marker of subclinical atherosclerotic disease and an independent predictor of cardiovascular morbidity and mortality, but also exhibits a dose–response relationship where the risk of CVD mortality increases proportionally with the severity of AAC scores (12). In order to access AAC, the Kauppila AAC score ranged from 0 to 24, developed by Kauppila and colleagues (13), is a quantifiable methodology utilizing lateral lumbar radiographs to assess abdominal aortic calcification. AAC score > 6 was used as a commonly reported cut-off point for severe AAC has been extensively defined in prior investigations (14, 15) and has been shown to forecast all-cause mortality and CVD mortality independently. In a longitudinal cohort study of 396 Japanese hemodialysis patients spanning from 2005 to 2014, distinct trajectories of abdominal aortic calcification were identified through group-based modeling, demonstrating that rapid nonlinear progression (adjusted HR 1.91, 95%CI 1.02–3.58) and advanced baseline with slow progression (adjusted HR 2.79, 95%CI 1.44–5.11) were independent predictors of heightened all-cause mortality risk when contrasted with stable patterns (16). Furthermore, there was a notable association between AAC progression and the development of CVD and myocardial infarction, with HR of 1.6 and 2.1, respectively (17). Additionally, there is a potential link between AAC and lower bone mineral density, increased fracture risk, and a decline in handgrip strength in the older adult (18, 19). Therefore, prevention and early detection of AAC, to slow its progression, are worth exploring and may be benefit to the patients.

The extension of human lifespan is intricately connected to the concomitant rise in chronic comorbidities and frailty syndromes, significantly impacting healthcare systems (20). Frailty, a geriatric syndrome characterized by diminished physiological capacity and increased vulnerability to stressors, susceptibility to stress, and impaired ability to maintain homeostasis, leading to negative patient outcomes (21). Due to the multiple multisystem dysfunctions associated with frailty, there is currently no single diagnostic tool or biomarker available for identifying and evaluating frailty. At present, the most widely used principal models to operationalize the frailty concept are the frail phenotype (22) (assessed by physical performances) and the frailty index (23) (assessed by multidimensional checklists encompassing biological, psychological, and sociological aspects). Frailty is highly common among the older adult population, substantially increasing the risk of negative health outcomes such as fall episodes, hospitalization, functional impairment, major CVD events and CVD mortality (24, 25). While frailty is also a reversible state, and decreased CVD risks were observed in individuals who reversed their frailty status (26). The multifactorial pathogenesis of frailty emerges through the convergence of age-related biological cascades, principally involving dysregulated neuroplasticity, endocrine axis disruption, persistent pro-inflammatory states, and tissue-specific epigenetic remodeling, which collectively drive systemic vulnerability in older populations (27, 28). Central nervous system dysfunction and musculoskeletal degeneration are considered key pathological events during the course of frailty (27), however, the link between cardiovascular disease (CVD) and frailty has garnered increased attention due to the acknowledgment that the aging process accelerates the onset and progression of age-related illnesses (29). Studies on the association between cardiovascular disease and frailty have mainly focused on endpoint events, with few studies reporting the association between frailty and subclinical atherosclerotic events. Although the FI is associated with various chronic diseases, the relationship between the FI and AAC and its potential clinical value are still unclear.

To fill the knowledge gap, the purpose of this study is to investigate the association between FI and AAC among middle-aged and older adults in US using the 2013–2014 National Health and Nutrition Examination Survey (NHANES) dataset. Through this study, the FI may become a valuable clinical indicator for early detection and managing subclinical atherosclerosis in clinical practice.

## 2 Methods

### 2.1 Participants and study design

**Figure 1:**
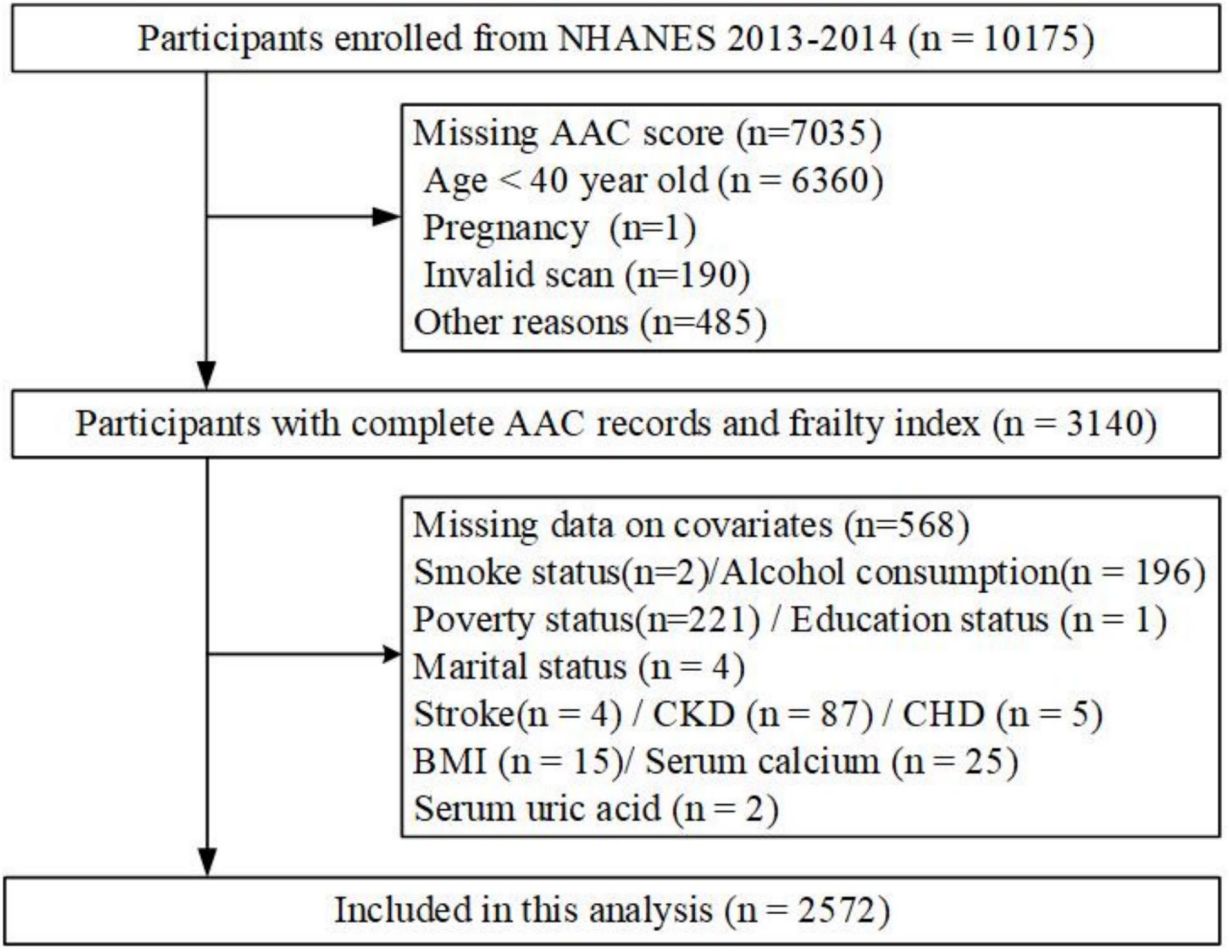
Flowchart illustrating the selection process of study participants.

This population-based cross-sectional study was conducted using data from the NHANES project, overseen by the National Center for Health Statistics at the Centers for Disease Control and Prevention (CDC) in the United States. The Institutional Review Board of the National Center of Health Statistics approved the initial survey protocol. All participants provided an informed consent form and complied with the Declaration of Helsinki (30). All NHANES data used in this analysis are publicly available at https://www.cdc.gov/nchs/nhanes. Since the dual-energy X-ray absorptiometry (DXA) scan was only performed during 2013–2014 in NHANES, participants from this period were included (31). After excluding participants under 40 years old (*n* = 6,360), those who were pregnant (*n* = 3), had invalid scans (*n* = 190), or were not scanned for other reasons (*n* = 482), 3,140 participants with complete AAC data were included. Additional exclusion criteria were missing data on covariates: smoking status (*n* = 2), alcohol consumption (*n* = 196), poverty status (*n* = 221), education status (*n* = 1), marital status (*n* = 4), stroke (*n* = 4), chronic kidney disease (CKD) (*n* = 87), coronary heart disease (CHD) (*n* = 5), body mass index (BMI) (*n* = 15), serum calcium (*n* = 25), and serum uric acid (*n* = 2). Ultimately, 2,572 adult participants were included in the primary analysis (Figure 1).

### 2.2 Measurement of frailty index

We assessed frailty using the frailty index (FI) approach proposed by Rockwood et al. The 49-item FI used in previous studies covered different systems, including cognition (Memory lapses and cognitive challenges), dependence (difficulties in performing daily activities), depression (assessed using the PHQ-9), comorbidities (various chronic conditions), hospital utilization and general health, physical performance, body assessment (grip power and BMI), and laboratory data (including blood counts and glucose levels), constructed according to standardized procedures published earlier (23, 32). Scores were aggregated and then normalized by dividing the total by the number of items. A scoring system ranging from 0 to 1 was established to enable the amalgamation of continuous and categorical variables, aligning with the severity of the deficit (Supplementary Table S1). To facilitate analysis, we transformed this continuous score into a categorical variable based on cutoffs established in previous literature (33, 34). We categorized our sample into three groups: non-frail (FI ≤ 0.15), pre-frail (0.15 < FI ≤ 0.25), and frail (FI > 0.25). All items of FI are shown in. A detailed summary of the variables comprising the frailty index along with their respective scores can be found in Supplementary Table S1.

### 2.3 Abdominal aortic calcification

Each participant’s scan and phantom scan were analyzed by UCSF using standard radiologic techniques and study specific NHANES protocols. The severity of abdominal aorta calcification was quantified using the AAC score. It was calculated based on Kauppila score system (13) by dual-energy X-ray absorptiometry (DXA, Densitometer Discovery A, Hologic, Marlborough, MA, United States) for each participant. The Kauppila scoring method involved dividing the anterior and posterior aortic walls into four segments, corresponding to the areas in front of the lumbar vertebrae L1–L4. Scores were obtained separately for these walls, resulting in a range from 0 to 6 for each vertebral level and 0 to 24 for the total score. Higher AAC scores indicated more severe calcification in the abdominal aorta. An AAC score greater than 6 was used as a commonly reported cut-off point for severe abdominal aortic calcification in previous study (35, 36). Both AAC score and severe AAC were included as outcome variables in our analysis.

### 2.4 Demographic characteristics and other covariate

In the analysis, we included self-reported data on age, sex, education, race, alcohol consumption, smoking status, and medical history. Race was classified into non-Hispanic Black, non-Hispanic White, Mexican American, and Multiracial/other races. Education level was simplified into three categories: below high school (less than 11th grade), high school graduate or General Educational Development (GED), and some college or higher (Associate’s degree or above). Additionally, marital status was grouped as either married or living with a partner, or as widowed/divorced/separated/never married. The Poverty-Income Ratio (PIR), an index of income relative to federally established poverty thresholds that accounts for economic inflation and family size, was estimated by dividing monthly family income by the poverty level (37). PIR was then classified into three groups: <1.30, 1.31 to 3.50, and >3.50. Nicotine exposure, alcohol use, and histories of diabetes, coronary heart disease (CHD), hypertension, stroke, and chronic kidney disease (CKD) were obtained via self-report questionnaires. Smoking status was assessed and classified as “never smoker” for individuals who smoked less than 100 cigarettes in their lifetime, “former smoker” for those who had smoked more than 100 cigarettes but currently do not smoke, and “current smoker” for individuals who smoked more than 100 cigarettes in their lifetime and smoke some days or every day (38, 39). Alcohol users were categorized into the following groups based on a previous study (40): “non-drinks,” “ex-drinks,” “current-drinks.” Diabetes was defined as meeting one of the following criteria: (1) self-reported diagnosis of diabetes by a doctor, (2) taking hypoglycemic drugs, (3) glycated hemoglobin (HbA1c) ≥ 6.5%, or (4) fasting blood glucose ≥ 7.0 mmol/L. Similarly, hypertension was defined as the self-reported diagnosis of hypertension by a doctor, taking antihypertensive medications, or an average systolic blood pressure ≥ 140 mmHg or average diastolic blood pressure ≥ 90 mmHg. Stroke was diagnosed among those who responded “yes” to the self-reported stroke question (“Has a doctor or other health professional ever told you that you had a stroke?”) (39). CHD was diagnosed among individuals responding “yes” to the self-reported CHD question (“Has a doctor or other health professional ever told you that you had coronary heart disease?”). CKD was diagnosed among individuals responding “yes” to the self-reported CKD question (“Has a doctor or other health professional ever told you had weak/failing kidneys?”). Height and weight were collected at the mobile examination center (MEC), and BMI was calculated using the formula: body weight (kg)/height squared (m^2^). Clinical indicators such as serum total calcium, serum phosphorus, serum alkaline phosphatase, serum creatinine, serum uric acid, triglycerides (TG), total cholesterol (TC), low-density lipoprotein cholesterol (LDL-C), and high-density lipoprotein cholesterol (HDL-C) were measured in the NHANES laboratory.

### 2.5 Statistical analysis

For continuous variables that met normal distribution, the mean ± standard deviation (SD) was used for statistical description, and an independent samples *t*-test was used for inter-group comparison. If the variables did not meet normal distribution, the median (Q1, Q3) was used for description, and the rank sum test was used for inter-group comparison. For categorical data, the number of cases (%) was used for description, and the chi-square test was used for comparison between groups. Fisher’s exact probability was employed when the chi-square test assumptions were not satisfied. Continuous data were compared using the Kruskal-Wallis test or one-way analysis of variance (ANOVA), and categorical data were compared using the chi-squared test. Covariates selected according to previous research (15, 41) and clinical expertise, which could potentially influence the relationship between FI and AAC, were further added to the multivariate Cox model. We used four levels of adjustment. Model 1, no covariates were adjusted. Model 2 was adjusted for age and sex. Model 3 was adjusted for age, sex, race and ethnicity, poverty income ratio (categorical), marital status, and education levels. Model 4 was adjusted for age, sex, race and ethnicity, poverty income ratio (categorical), marital status, education levels, hypertension, body mass index, total cholesterol, serum total calcium, serum phosphorus, serum creatinine, and serum alkaline phosphatase. We further conducted sensitivity analysis after categorizing the FI group to evaluate robustness. To facilitate the interpretation of regression coefficients (42, 43), FI was converted to FI Z-score using Z-score transformations for further analysis. A generalized additive model (GAM) and smooth curve fittings were employed to address nonlinearity between FI Z-score and AAC. When a non-linear correlation was observed, a two-piecewise linear regression model (segmented regression model) was used to fit each interval and calculate the threshold effect. A log-likelihood ratio test comparing the one-line model (non-segmented) to the two-piecewise linear regression model was conducted to determine the existence of a threshold. The breakpoint (K) was further determined by a two-step recursive method (39).

Additionally, subgroup analysis of the associations between FI Z-score and AAC (AAC score and Severe AAC) was conducted using stratified multivariable logistic regression models. Stratified factors included sex, age (40–65, and ≥65 years), DM, CHD, CKD, PIR (<1.30, 1.31–3.50, >3.50), Marital Status (status 1: married or living with a partner, status 2:widowed/divorced/separated/never married) which were also treated as potential effect modifiers. An interaction term was added to evaluate heterogeneity using the likelihood ratio test. All analyses were performed using R version 4.1.3 (http://www.R-project.org, The R Foundation) and Empower software (www.empowerstats.com; X&Y Solutions, Inc., Boston, MA). Statistical significance was considered at a two-sided *p* < 0.05.

## 3 Results

### 3.1 Baseline characteristics of the study population

Table 1 shows that a total of 2,572 participants were enrolled in this study, with an average age of 58.95 ± 11.95 years, including 1,244 males (48.37%). The prevalence of the non-frail state (FI ≤ 0.15), pre-frail state (0.15 < FI ≤ 0.25), and frail state (FI > 0.25) was 53.97, 29.24, and 17.61%, respectively. The median AAC score was 0 (Q1, Q3: 0.00–2.00) in the overall cohort, showing progressive increases across FI categories [non-frail: 0 (Q1, Q3: 0.00–1.00); pre-frail:0.00 (Q1, Q3: 0.00–2.00); frail: 0 (Q1, Q3: 0.00–3.00), *p* < 0.001]. The overall prevalence of severe AAC was 11.12%, and it also increased with the FI index (non-frail: 7.13%; pre-frail: 14.34%; frail: 18.20%; *p* < 0.001). Those with higher FI were notably older, more likely to be female, non-Hispanic white, below high school education, with a poverty ratio < 1.3, and never married, widowed, divorced, or separated (all *p* values < 0.05). Furthermore, individuals with a history of hypertension, diabetes, stroke, heart disease, or CKD had higher FI (all *p* values < 0.05). Participants with higher FI displayed higher BMI, serum phosphorus, serum uric acid, serum alkaline phosphatase, serum creatinine, and triglycerides, but lower total cholesterol, HDL-C, and LDL-C (all *p* values < 0.05). However, there was no significant difference in serum total calcium (*p* > 0.05) (Table 1).

**Table 1:** Baseline characteristics of study population according frailty status.

Characteristics	Total *n* = 2,572	Non-frail *n* = 1,388	Pre-frail *n* = 739	Frail *n* = 445	*P* value
Age, years, mean (SD)	58.95 (11.95)	56.75 (11.25)	60.80 (12.33)	62.77 (11.95)	<0.001
Male, no (%)	1,244 (48.37)	739 (53.24)	349 (47.23)	156 (35.06)	<0.001
Race/ethnicity, no (%)					<0.001
Non-Hispanic Black	490 (19.05)	217 (15.63)	180 (24.36)	93 (20.90)	
Non-Hispanic White	1,197 (46.54)	647 (46.61)	331 (44.79)	219 (49.21)	
Mexican American	319 (12.40)	173 (12.46)	93 (12.58)	53 (11.91)	
Multiracial/other	566 (22.01)	351 (25.29)	135 (18.27)	80 (17.98)	
Education level, no (%)					<0.001
Below high school	537 (20.88)	233 (16.79)	165 (22.33)	139 (31.24)	
High school graduate or GED	585 (22.74)	294 (21.18)	177 (23.95)	114 (25.62)	
Some college or above	1,450 (56.38)	861 (62.03)	397 (53.72)	192 (43.15)	
Poverty ratio, no (%)					<0.001
<1.3	757 (29.43)	318 (22.91)	221 (29.91)	218 (48.99)	
1.3–3.5	897 (34.88)	456 (32.85)	290 (39.24)	151 (33.93)	
>3.5	918 (35.69)	614 (44.24)	228 (30.85)	76 (17.08)	
Marital status					<0.001
Married or living with partner	1827 (71.03)	1,070 (77.09)	508 (68.74)	249 (55.96)	
Never married/widowed/divorced/separated	745 (28.97)	318 (22.91)	231 (31.26)	196 (44.04)	
Smoking status, no (%)					<0.001
Never	1,362 (52.95)	821 (59.15)	359 (48.58)	182 (40.90)	
Former	735 (28.58)	354 (25.50)	233 (31.53)	148 (33.26)	
Current	475 (18.47)	213 (15.35)	147 (19.89)	115 (25.84)	
Alcohol consumption, no (%)					<0.001
Non-drinks	365 (14.19)	183 (13.18)	113 (15.29)	69 (15.51)	
Ex-drinks	531 (20.65)	234 (16.86)	162 (21.92)	135 (30.34)	
Current-drinks	1,676 (65.16)	971 (69.96)	464 (62.79)	241 (54.16)	
History of hypertension, no (%)	1,391 (54.08)	557 (40.13)	482 (65.22)	352 (79.10)	<0.001
History of DM, no (%)	606 (23.56)	167 (12.03)	245 (33.15)	194 (43.60)	<0.001
History of stroke, no (%)	109 (4.24)	12 (0.86)	33 (4.47)	64 (14.38)	<0.001
History of heart disease, no (%)	136 (5.29)	10 (0.72)	54 (7.31)	72 (16.18)	<0.001
History of CKD, no (%)	570 (22.16)	191 (13.76)	205 (27.74)	174 (39.10)	<0.001
Body mass index, kg/m2, mean (SD)	28.55 (5.60)	27.66 (5.09)	29.28 (5.79)	30.11 (6.25)	<0.001
Serum total calcium, mmol/L, mean (SD)	2.36 (0.09)	2.36 (0.09)	2.37 (0.09)	2.36 (0.11)	0.098
Serum phosphorus, mmol/L, mean (SD)	4.05 (0.37)	4.02 (0.33)	4.06 (0.39)	4.09 (0.46)	0.001
Serum uric acid, μmol/L, mean (SD)	324.59 (82.42)	320.46 (76.20)	326.93 (84.77)	333.60 (95.42)	0.009
Serum alkaline phosphatase, median (Q1, Q3)	64.0 (53.0–79.0)	62.0 (51.0–75.0)	65.0 (54.0–79.0)	70.0 (57.0–84.0)	<0.001
Serum creatinine, μmol/L, median (Q1, Q3)	77.79 (65.42–92.82)	77.79 (65.42–89.28)	77.79 (64.97–94.59)	81.33 (67.18–101.66)	<0.001
Total cholesterol (mmol/L), mean (SD)	5.06 (1.14)	5.19 (1.08)	4.95 (1.10)	4.83 (1.29)	<0.001
Triglycerides (mmol/L), median (Q1, Q3)	1.13 (0.77–1.67)	1.08 (0.74–1.49)	1.15 (0.78–1.69)	1.35 (0.87–2.00)	<0.001
HDL, median (Q1, Q3)	1.32 (1.09–1.63)	1.37 (1.11–1.68)	1.29 (1.06–1.60)	1.24 (1.03–1.58)	<0.001
LDL, median (Q1, Q3)	2.92 (2.30–3.54)	3.00 (2.40–3.60)	2.82 (2.28–3.53)	2.61 (1.94–3.23)	<0.001
Frailty index, median (Q1, Q3)	0.14 (0.09–0.21)	0.09 (0.07–0.12)	0.19 (0.17–0.21)	0.31 (0.28–0.37)	<0.001
AAC Score, median (Q1, Q3)	0.00 (0.00–2.00)	0.00 (0.00–1.00)	0.00 (0.00–2.00)	0.00 (0.00–4.00)	<0.001
Severe AAC^‡^, no (%)	286 (11.12)	99 (7.13)	106 (14.34)	81 (18.20)	<0.001

Data presented as mean (standard deviation, SD) or median (Q1, Q3) for continuous and no. (%) values for categorical.

ACC, Abdominal aortic calcification; GED, General educational development test; BMI, Body mass index; HDL, High-density lipoprotein; CKD, Chronic kidney disease; PIR, poverty income ratio.

^‡Severe AAC was determined by the total AAC score that exceeded 6.^

### 3.2 Relationship between FI and severe ACC

Table 2 shows the association between FI and severe AAC. We found that higher FI Z-score and FI were correlated with severe AAC in both unadjusted and adjusted models. After full adjustment (adjusted for age, sex, race and ethnicity, poverty income ratio, marital status, education levels, hypertension, body mass index, total cholesterol, serum total calcium, serum phosphorus, serum creatinine, and serum alkaline phosphatase), participants with a unit higher FI Z-score had a 20% increased risk of severe AAC (OR = 1.20, 95% CI 1.04–1.39). Participants with a unit higher FI had a 5.36-fold increased risk of severe AAC (OR = 6.36, 95% CI 1.48–27.41). When FI was categorized as non-frail, pre-frail, and frail, participants in the pre-frail status exhibited a significantly 0.41-fold increased likelihood compared to the non-frail status (OR = 1.41, 95% CI 1.02–1.97; *P* for trend = 0.038).

**Table 2:** Association between the frailty index and abdominal aortic calcification.

Variables	Model 1		Model 2		Model 3		Model 4	
AAC score, continuous	β (95%CI)	*P* value	β (95%CI)	*P* value	β (95%CI)	*P* value	β (95%CI)	*P* value
FI per SD increase	0.59 (0.45, 0.72)	<0.0001	0.30 (0.17, 0.43)	<0.0001	0.30 (0.16, 0.43)	<0.0001	0.26 (0.12, 0.41)	0.0003
FI	5.89 (4.55, 7.24)	<0.0001	3.05 (1.75, 4.34)	<0.0001	2.98 (1.64, 4.31)	<0.0001	2.64 (1.20, 4.08)	0.0003
FI								
Non-frail	0 (Reference)		0 (Reference)		0 (Reference)		0 (Reference)	
Pre-frail	0.95 (0.64, 1.26)	<0.0001	0.50 (0.21, 0.79)	0.0007	0.54 (0.25, 0.83)	0.0003	0.51 (0.21, 0.81)	0.0008
Frail	1.45 (1.08, 1.82)	<0.0001	0.79 (0.44, 1.15)	<0.0001	0.75 (0.38, 1.11)	<0.0001	0.65 (0.27, 1.03)	0.0008
*P* for trend	0.001		0.001		0.001		0.001	
**Sever AAC, category**	**OR (95%CI)**	***P* value**	**OR (95%CI)**	***P* value**	**OR (95%CI)**	***P* value**	**OR (95%CI)**	***P* value**
FI per SD increase	1.50 (1.35, 1.67)	<0.0001	1.26 (1.11, 1.43)	0.0003	1.25 (1.09, 1.42)	0.0009	1.20 (1.04, 1.39)	0.0130
FI	59.54 (20.23, 175.24)	<0.0001	10.21 (2.89, 36.10)	0.0003	9.25 (2.49, 34.43)	0.0009	6.36 (1.48, 27.41)	0.0130
FI								
Non-frail	1 (Reference)		1 (Reference)		1 (Reference)		1 (Reference)	
Pre-frail	2.18 (1.63, 2.91)	<0.0001	1.46 (1.07, 2.00)	0.0186	1.46 (1.06, 2.02)	0.0198	1.41 (1.02, 1.97)	0.0400
Frail	2.90 (2.11, 3.97)	<0.0001	1.71 (1.21, 2.42)	0.0023	1.65 (1.15, 2.36)	0.0066	1.46 (0.99, 2.15)	0.0572
*P* for trend	0.001		0.001		0.004		0.038	

ACC Abdominal aortic calcification; BMI, body mass index; AKP, serum alkaline phosphatase; OR, odds ratio; CI, 95% confidence interval; β, effect size.

Model 1: no covariates were adjusted.

Model 2: adjusted for age, sex.

Model 3: adjusted for age, sex, race and ethnicity, poverty income ratio (category), marital status, education levels.

Model 4: adjusted for age, sex, race and ethnicity, poverty income ratio (category), marital status, education levels, hypertension, body mass index, total cholesterol, serum total calcium, serum phosphorus, serum creatinine, serum alkaline phosphatase.

**Figure 2:**
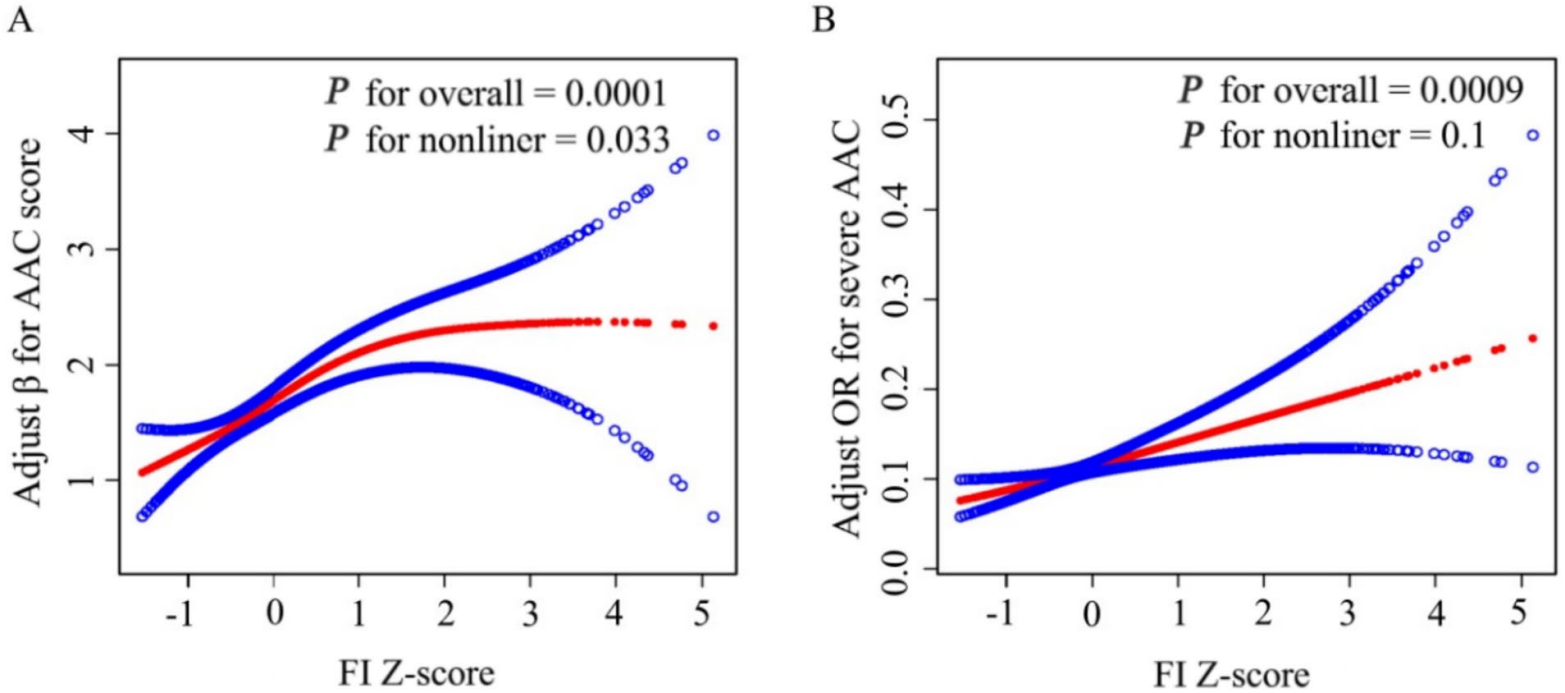
Smooth Curve Fitting Detected A nonlinear positive relationship between FI-Z score and AAC score **(A)** and a Liner relationship between FI Z-score and Sever AAC **(B)** by the generalized additive model. β/OR (Red lines) and 95% CI (under the blue curve areas) were adjusted for age, sex, race and ethnicity, poverty income ratio (category), marital status, education levels, hypertension, body mass index, total cholesterol, serum total calcium, serum phosphorus, serum creatinine, serum alkaline phosphatase.

In addition, a linear relationship was detected according to the results of smooth curve fitting between FI and severe AAC after full adjustment (*p* value for LRT test = 0.1) [adjusted for age, sex, race and ethnicity, poverty income ratio (categorical), marital status, education levels, hypertension, body mass index, total cholesterol, serum total calcium, serum phosphorus, serum creatinine, and serum alkaline phosphatase] (Figure 2B; Table 3).

**Table 3:** Threshold effect analysis of FI Z-Score on AAC using a two-piecewise linear regression model.

Outcomes	Severe AAC		AAC score	
	OR (95%CI)	*P* value	β (95%CI)	*P* value
FI Z-score				
Fitting by standard linear model	1.27 (1.10, 1.46)	0.0009	0.33 (0.19, 0.47)	<0.0001
Fitting by two-piecewise linear model				
Turn point (K)	0.52		0.78	
FI Z-score < K	1.58 (1.17, 2.13)	0.0026	0.52 (0.30, 0.75)	<0.0001
FI Z-score > K	1.08 (0.84, 1.38)	<0.5522	0.04 (−0.25, 0.34)	0.7725
*P* for Log-likelihood ratio	0.1		**0.033**	

95% CI, 95% confidence interval. OR, Odds Ratio. Bold font indicates that the log likelihood ratio test is statistically significant (*p* < 0.05). Adjusted for age, sex, race and ethnicity, poverty income ratio (category), marital status, education levels, hypertension, body mass index, total cholesterol, serum total calcium, serum phosphorus, serum creatinine, serum alkaline phosphatase.

### 3.3 A nonlinear relationship between FI and AAC score

FI was positively associated with the likelihood of a higher AAC score with statistical significance, and this association remained stable across our four models. After full adjustment, each unit increase in FI Z-score was associated with a 0.26 unit increase in AAC score (β = 0.26, 95% CI: 0.12–0.41). Each unit increase in FI was associated with a 1.64-fold increase in AAC score (β = 2.64, 95% CI: 1.20–4.08). Sensitivity analysis was conducted with frailty status as a categorical variable (non-frail, pre-frail, frail). In the fully adjusted model, compared with the non-frail status, the adjusted β for participants in pre-frail status and frail status were 0.51 and 0.65, respectively (Pre-frail: β = 0.51, 95% CI: 0.21–0.81; Frail: β = 0.65, 95% CI: 0.27–1.03; *P* for trend = 0.0001) (Table 2).

However, smooth curve fitting exhibited a non-linear relationship between FI and AAC score after full adjustment (*p* value for LRT test < 0.05) [adjusted for age, sex, race and ethnicity, poverty income ratio (categorical), marital status, education levels, hypertension, body mass index, total cholesterol, serum total calcium, serum phosphorus, serum creatinine, and serum alkaline phosphatase] (Figure 2A). Then, FI was converted to FI Z-score to analysis the dose-relationship. A generalized additive model (GAM) and smooth curve fittings were employed to address nonlinearity between FI Z-score and AAC. We further calculated the turning point (K) to be 0.78. On the left of the turning point, a positive relationship between FI Z-score and AAC score (β = 0.52, 95% CI: 0.30–0.75; *p* < 0.0001) was detected. However, no statistically significant relationship between FI Z-score and AAC score was found on the right of the turning point (β = 0.04, 95% CI: −0.25–0.34; *p* = 0.7725) (Table 3).

### 3.4 Subgroup analysis and interaction test

**Figure 3:**
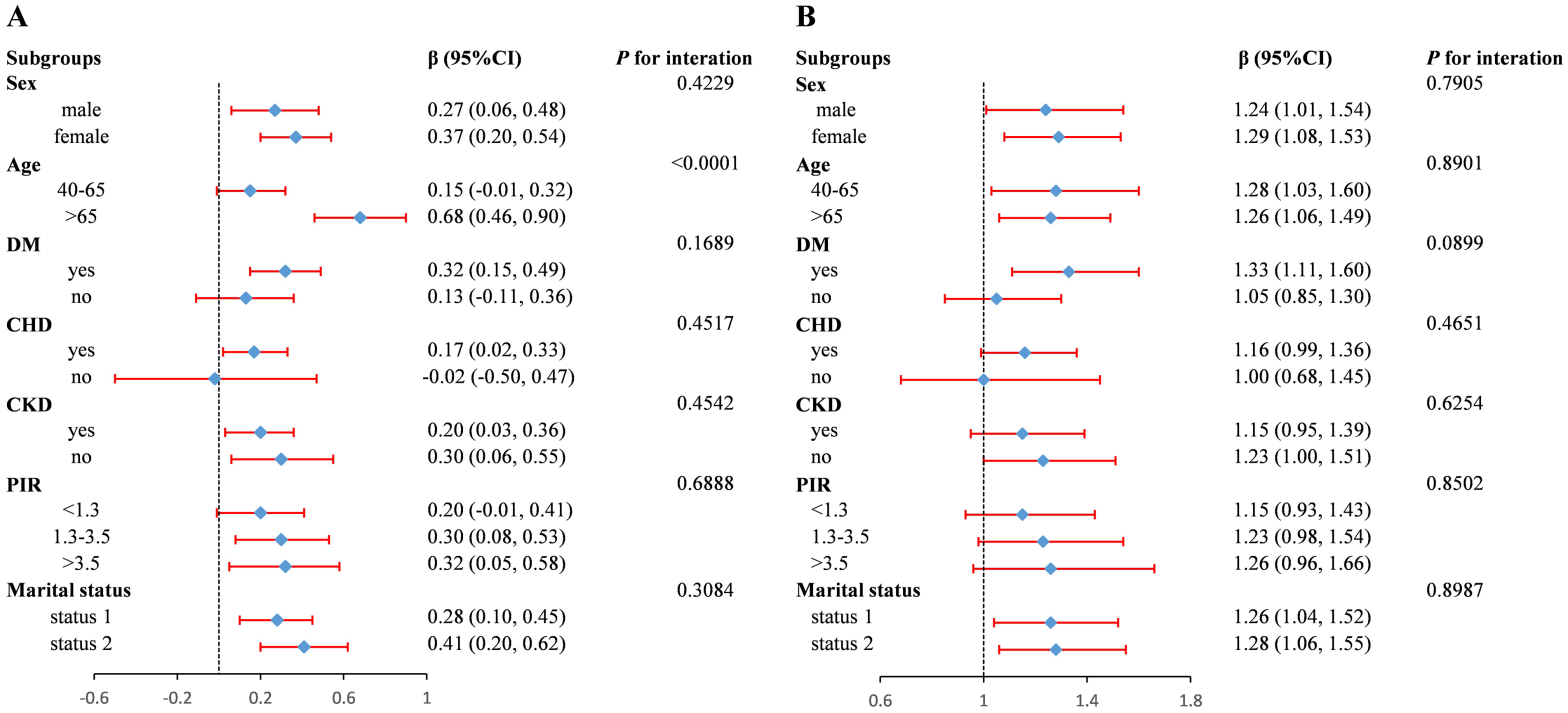
Forest plot of subgroup analysis for the association between FI Z-score and AAC score **(A)** and Severe AAC **(B)**. Adjusted for age, sex, race and ethnicity, poverty income ratio (category), marital status, education levels, hypertension, body mass index, total cholesterol, serum total calcium, serum phosphorus, serum creatinine, serum alkaline phosphatase, except for the subgroup variable. OR, odds ratio; 95% CI, 95% confidence interval. Status 1: married or living with a partner; Status 2: widowed/divorced/separated/never married.

To evaluate whether the association between FI Z-score and AAC was consistent in the overall population and to identify potential differences among subgroups, we conducted subgroup analysis and interaction tests stratified by sex, age, DM, CHD, CKD, PIR, and marital status. In the subgroup analysis, the association between FI Z-score and AAC score was not consistently significant across certain groups (Figure 3A). Interaction testing indicated that gender, DM, CHD, CKD, PIR, and marital status did not significantly impact the association between FI and AAC score (all *P* for interaction > 0.05). However, the age subgroup significantly influenced this association (*P*-interaction < 0.05). The positive association between FI and AAC score appeared stronger in populations older than 65 years (β per 1 SD increase, 0.68; 95% CI: 0.46–0.90). We also found that FI Z-score was positively associated with severe AAC in subgroups stratified by gender (male or female), history of DM and CHD, CKD (yes or no), PIR > 1.3, and marital status. For severe AAC, we detected no statistical significance for gender, age, DM, CHD, CKD, PIR, and marital status (*P*-interaction > 0.05) (Figure 3B).

## 4 Discussion

In this cross-sectional study with a total of 2,572 subjects, we observed a positive correlation between the FI and AAC. Whether AAC was considered a continuous or categorical variable, the FI was found to be an independent risk factor for AAC. Interestingly, we found that the FI Z-score was linearly related to the occurrence of severe AAC through smoothing curves, while it was nonlinearly related to the AAC score. Different relationships of FI Z-score on AAC score were detected on the left and right sides of the breakpoint. FI Z-score was positively associated with the likelihood of AAC score on the left side of breakpoint (*K* = 0.78), while the association on the right of breakpoint was of no statistical significance. Our findings demonstrate a significant association between FI and AAC, suggesting FI may serve as a correlational biomarker requiring further validation of its temporal relationship with AAC pathogenesis.

To our knowledge, this is the first study to examine the relationship between FI and AAC, highlighting a direct link between FI severity and increased subclinical cardiovascular risk. The Frailty has been consistently associated with higher risks of multiple adverse health outcomes, including loss of ability to carry out daily activities (ADL), falls and fractures, disability, multiple chronic diseases, hospitalization, and even death. Additionally, a prospective cohort study of the Chinese population showed that the FI was associated with all-cause and cause-specific mortality, independent of the actual age of both young and older adult individuals (44). Among 2,894 adults diagnosed with type 2 diabetes mellitus (T2DM) in the NHANES study, a positive linear relationship was observed between higher FI and an increased risk of congestive heart failure (OR = 3.60, 95CI%: 2.34–5.54). Furthermore, individuals with higher FI exhibited an 86% higher risk of all-cause mortality (HR = 1.86, 95%CI:1.55–2.24) and a 66% elevated risk of cardiovascular mortality (HR = 1.66, 95%CI:1.18–2.33) in comparison to non-frail patients. A meta-analysis has shown that a genetically determined higher FI is associated with an odds ratio of 1.46 for coronary artery disease, 1.62 for myocardial infarction, and 1.46 for heart failure (45). Emerging evidence demonstrates that reversing frailty status significantly reduces the risk of cardiovascular events, underscoring the critical need to integrate frailty assessment into preclinical risk stratification (26). The Multi-Ethnic Study of Atherosclerosis (MESA) revealed that AAC exhibited a more robust association with cardiovascular mortality and all-cause mortality than CAC, indicating its earlier appearance in the progression of atherosclerosis. Thus, AAC emerges as a more clinically relevant and sensitive early biomarker of systemic atherosclerosis (46). Existing studies have mainly examined the link between frailty and clinical outcomes. However, the connection between the frailty index (FI) and subclinical cardiovascular conditions, specifically AAC as an early indicator of atherosclerosis, lacks thorough investigation. Moreover, Lin CH’ s research report that a significant association between frailty and subclinical peripheral arterial disease (measured by the Ankle-Brachial Index), and this relationship remains significant even when considering age, blood pressure, and blood lipid levels. Subsequently, The Multicenter AIDS Cohort Study (MACS) reveals that frailty is independently associated with subclinical coronary atherosclerosis in HIV-uninfected men, but not in HIV-infected men. In our study we also found that the FI was positively correlated with AAC and we observe a linear positive correlation between the FI and severe AAC in both unadjusted and adjusted models. Our study broadens existing knowledge by extending the findings to middle-aged cohorts and using a nationally representative sample from the United States. Additionally, we applied a more accurate and commonly used approach to define frailty, specifically the frailty index, which includes up to 49 items for assessment. Collectively, these findings indicate that the FI could function as a valuable biomarker for early identification of subclinical atherosclerosis and prediction cardiovascular events, as demonstrated in our research. Incorporating FI into routine screenings for symptom-free adults could improve preventive healthcare strategies.

The potential mechanisms of this positive association between FI and AAC has not been fully understood. Frailty is associated with diminished antioxidant defense and elevated oxidative stress markers, hastening the progression from prefrailty to frailty (47).Elevated inflammatory markers (such as TNF-α, IL-1β, IL-1, IL-6, and IL-18) and pro-inflammatory cytokines contribute to frailty by promoting protein catabolism (48, 49). The release of inflammatory mediators leads to endothelial dysfunction and vascular wall degradation, which increases the likelihood of AAC (50). Previous studies have also indicated that inflammatory mediators can activate vascular smooth muscle cell calcification through various pathways, leading to the occurrence and development of AAC (51, 52). Additionally, some studies have shown that the systemic immune-inflammatory index (SII), a new inflammation assessment indicator, is positively correlated with AAC. This association is more significant in the older adult and has potential value in identifying the severity of AAC (53). In addition to elevated levels of inflammation, frail older adult individuals often experience disturbances in calcium/phosphate metabolism and decreased bone mineral density, which are important causes of VC (54). Epidemiological studies have also reported an association between AAC and osteoporosis, accelerated bone loss, and vertebral and hip fractures (55). Notably, increased oxidative stress and oxidative stress-induced signaling are common features in frail populations (56), studies have shown that elevated intracellular oxidative stress, through oxidized lipids and H_2_O_2_, induces VSMC osteogenic differentiation and calcification (57). In chronic kidney disease patients, increased systemic oxidative stress is a key feature of the uremic state (58), which is highly correlated with increased VC (59). In summary, inflammatory response, metabolic imbalance, oxidative stress, and other factors are common pathophysiological changes in individuals with a high FI, and these changes trigger the occurrence of VC, including AAC, ultimately increasing the incidence and mortality of cardiovascular disease. Therefore, these studies explain the association between FI and AAC at the molecular mechanism level.

Although our study utilized a complex multi-stage probability sampling design, which enhanced the reliability and representativeness of our findings, we ultimately identified a significant positive association between FI and AAC. The findings of this study could provide a valuable insight for the management and prevention of subclinical atherosclerotic events. However, our study also possesses several potential limitations. Firstly, the cross-sectional design of this study prevented the establishment of causal relationships between FI and AAC. Future longitudinal, experimental studies or Mendelian randomization are crucial for elucidating these causal relationships, investigating whether the FI raises the risk of developing AAC or if the presence of AAC contributes to the deterioration of FI. Secondly, this study utilized self-reported retrospective data resulting in unavoidable recall bias and social desirability bias. Owing to the absence of data on these variables in NHANES, which are not included in the original dataset, thus constraining our inclusion of these covariates in the analysis. Thirdly, our study, like other observational studies, could not eliminate residual or unknown confounding factors or accidental confounding effects resulting from measurement errors and unmeasured variables such as psychosocial stress or genetic susceptibility. Future research is essential to explore the relationship and biological mechanisms between FI and AAC by incorporating advanced technologies such as machine learning and multi-omics approaches. Fourthly, NHANES was a study conducted in US, thus we could only evaluate the association between FI and AAC in US adults, the sample’s geographical and demographic specificity may restrict generalizability to other populations.

Our study has important clinical and public health implications. The findings of this study emphasize the notable link between FI and AAC, emphasizing the need for targeted clinical strategies to assess and manage frailty in older adults. By integrating FI assessments into routine clinical evaluations, healthcare providers can enhance early identification of individuals at elevated risk for subclinical atherosclerosis, ultimately promoting better cardiovascular health outcomes in aging populations. These investigations may ultimately contribute to the development of effective prevention and intervention aimed at mitigating the impact of subclinical atherosclerosis in vulnerable populations.

## 5 Conclusion

This cross-sectional study demonstrated that elevated FI levels were associated with higher severe AAC and a nonlinear positive relationship with AAC score, indicating that FI may serve as a biomarker for early subclinical atherosclerosis detection in US adults aged ≥ 40 years. Additionally, high-quality studies are necessary to validate our findings and establish a causal link between the two variables.

## Data Availability

All NHANES data used in this analysis are publicly available at https://www.cdc.gov/nchs/nhanes.
