# Supplementary material for: The association between frailty index and abdominal aortic calcification in the middle-aged and older US adults: NHANES 2013–2014
Source: Front Public Health. 2025 May 7;13:1546647. doi: 10.3389/fpubh.2025.1546647 (PMC12092342; doi:10.3389/fpubh.2025.1546647)
Supplement: Supplementary file 1 [file Table_1.docx]

Table S1. Variables in the 49-item frailty index and their respective scorings.

| **Cognition** |  |
| --- | --- |
| 1.Experience confusion/memory problems | Yes=1, No=0 |
| **Dependence** |  |
| 2.Managing money | Difficulty=1, No Difficulty=0 |
| 3.Stooping, crouching, kneeling | Difficulty=1, No Difficulty=0 |
| 4.Lifting or carrying | Difficulty=1, No Difficulty=0 |
| 5.House chore | Difficulty=1, No Difficulty=0 |
| 6.Preparing meals | Difficulty=1, No Difficulty=0 |
| 7.Standing up from armless chair | Difficulty=1, No Difficulty=0 |
| 8.Getting in and out of bed difficulty | Difficulty=1, No Difficulty=0 |
| 9.Using fork, knife, drinking from cup | Difficulty=1, No Difficulty=0 |
| 10.Dressing yourself | Difficulty=1, No Difficulty=0 |
| 11.Standing for long periods difficulty | Difficulty=1, No Difficulty=0 |
| 12.Grasp/holding small objects | Difficulty=1, No Difficulty=0 |
| 13.Attending social event | Difficulty=1, No Difficulty=0 |
| 14.Push or pull large objects | Difficulty=1, No Difficulty=0 |
| 15.walking for a quarter mile difficulty | Difficulty=1, No Difficulty=0 |
| 16.walking up 10 steps difficulty | Difficulty=1, No Difficulty=0 |
| **Depressive Symptoms** |  |
| 17.Have little interest in doing things | Nearly every day=1, More than half the days=0.66, Several days=0.33, Not at all=0 |
| 18.Feeling down, depressed, or hopeless | Nearly every day=1, More than half the days=0.66, Several days=0.33, Not at all=0 |
| 19.Trouble sleeping or sleeping too much | Nearly every day=1, More than half the days=0.66, Several days=0.33, Not at all=0 |
| 20.Feeling tired or having little energy | Nearly every day=1, More than half the days=0.66, Several days=0.33, Not at all=0 |
| 21.Poor appetite or overeating | Nearly every day=1, More than half the days=0.66, Several days=0.33, Not at all=0 |
| 22.Feeling bad about yourself | Nearly every day=1, More than half the days=0.66, Several days=0.33, Not at all=0 |
| 23.Trouble concentrating on things | Nearly every day=1, More than half the days=0.66, Several days=0.33, Not at all=0 |
| **Comorbidities** |  |
| 24.Arthritis | Yes=1, Suspect=0.5, No=0 |
| 25.Thyroid problems | Yes=1, Suspect=0.5, No=0 |
| 26.Chronic bronchitis | Yes=1, Suspect=0.5, No=0 |
| 27.Cancer | Yes=1, Suspect=0.5, No=0 |
| 28.Congestive heart failure  29.Coronary heart disease | Yes=1, Suspect=0.5, No=0  Yes=1, Suspect=0.5, No=0 |
| 30.Angina | Yes=1, Suspect=0.5, No=0 |
| 31.Heart attack | Yes=1, Suspect=0.5, No=0 |
| 32.Stroke | Yes=1, Suspect=0.5, No=0 |
| 33.Blood pressure | Yes=1, Suspect=0.5, No=0 |
| 34.Diabetes  35.Weak/failing kidneys | Yes=1, Suspect=0.5, No=0  Yes=1, Suspect=0.5, No=0 |
| 36.Urinary leakage | Yes=1, Suspect=0.5, No=0 |
| **Hospital Utilization and Access to Care** |  |
| 37.Self-rated health | Fair, poor=1, Excellent, Very good, good=0 |
| 38.Health now compared 1 year ago | Worse=1, About the same, Better=0 |
| 39.Overnight hospital patient in past year | Yes=1, No=0 |
| 40.Frequency of healthcare using during past year | None=0, 1-5=0.5, 5 and more than 5=1 |
| 41.Number of prescribed medications | None=0, 1-4=0.5, More than 5=1 |
| **Physical Performance and Anthropometry** |  |
| 42.Body mass index  43.Handgrip Strength | <18.5, ≥30=1  25-<30=0.5  18.5-25=0  MALE: FEMALE:  For BMI≤24, GS≤29 For BMI≤23, GS≤17  For BMI 24.1-28, GS≤30 For BMI 23.1-26, GS≤17.3  For BMI＞28, GS≤32=1 For BMI26.1-29, GS≤18 |
| **Laboratory Values** | For BMI＞29, GS≤21=1 |
| 44. Glycohemoglobin (%)  45.Red blood cell count (million cells/μL) | 0%-5.7%=0,>5.7%=1  M: 4.7-6.1=0, Other=1; F: 4.2-5.4=0, Other=1 |
| 46.Hemoglobin (g/dL) | M:13.5-18=0, Other=1; F: 12-16=0, Other=1 |
| 47.Red cell distribution width (%) | 11.6-14.6=0, Other=1 |
| 48.Lymphocyte percent(%) | 20-40=0, Other=1 |
| 49.Segmented neutrophils percent(%) | 40-80=0, Other=1 |

BMI, Body mass index; GS, grip strength.
